# Circulating Tissue Factor-Positive Procoagulant Microparticles in Patients with Type 1 Diabetes

**DOI:** 10.2147/DMSO.S225761

**Published:** 2019-12-31

**Authors:** Chenghui Zhang, Qing Ou, Yan Gu, Gaiping Cheng, Rong Du, Li Yuan, Ruth LM Cordiner, Deying Kang, Jiaying Zhang, Qiaorong Huang, Chuan Yu, Li Kang, Xuan Wang, Xin Sun, Xianming Mo, Haoming Tian, Ewan R Pearson, Wentong Meng, Sheyu Li

**Affiliations:** 1 Department of Endocrinology and Metabolism, West China Hospital, Sichuan University, Chengdu 610041, People’s Republic of China; 2 Department of Endocrinology and Metabolism, Hospital of Chengdu Office of People’s Government of Tibetan Autonomous Region, Chengdu 610041, People’s Republic of China; 3 Department of Clinical Nutrition, West China Hospital, Sichuan University, Chengdu 610041, People’s Republic of China; 4 Division of Population Health and Genomics, Ninewells Hospital and School of Medicine, University of Dundee, Dundee DD1 9SY, Scotland, UK; 5 Chinese Evidence-Based Medicine Center, West China Hospital, Sichuan University, Chengdu 610041, People’s Republic of China; 6 Department of Ophthalmology, West China Hospital, Sichuan University, Chengdu 610041, People’s Republic of China; 7 Laboratory of Stem Cell Biology, State Key Laboratory of Biotherapy, West China Hospital, Sichuan University, Chengdu 610041, People’s Republic of China; 8 Department of Health-Related Social and Behavioral Science, West China School of Public Health, Sichuan University, Chengdu 610041, People’s Republic of China; 9 Division of Systems Medicine, Ninewells Hospital and School of Medicine, University of Dundee, Dundee DD1 9SY, Scotland, UK; 10 Science for Life Laboratory, Department of Medical Cell Biology, Uppsala University, Uppsala 75123, Sweden

**Keywords:** type 1 diabetes mellitus, microparticles, tissue factor, diabetic retinopathy

## Abstract

**Aim:**

To investigate the count of circulating tissue factor-positive (TF^+^) procoagulant microparticles (MPs) in patients with type 1 diabetes mellitus (T1DM).

**Methods:**

This case-control study included patients with T1DM and age and sex-matched healthy volunteers. The counts of phosphatidylserine-positive (PS^+^) MPs and TF^+^PS^+^MPs and the subgroups derived from different cell types were measured in the peripheral blood sample of the two groups using multicolor flow cytometric assay. We compared the counts of each MP between groups as well as the ratio of the TF^+^PS^+^MPs and PS^+^MPs (TF^+^PS^+^MPs/PS^+^MPs).

**Results:**

We recruited 36 patients with T1DM and 36 matched healthy controls. Compared with healthy volunteers, PS^+^MPs, TF^+^PS^+^MPs and TF^+^PS^+^MPs/PS^+^MPs were elevated in patients with T1DM (PS^+^MPs: 1078.5 ± 158.08 vs 686.84 ± 122.04/μL, *P* <0.001; TF^+^PS^+^MPs: 202.10 ± 47.47 vs 108.33 ± 29.42/μL, *P* <0.001; and TF^+^PS^+^MPs/PS^+^MPs: 0.16 ± 0.04 vs 0.19 ± 0.05, P = 0.004), mostly derived from platelet, lymphocytes and endothelial cells. In the subgroup analysis, the counts of total and platelet TF^+^PS^+^MPs were increased in patients with diabetic retinopathy (DR) and with higher HbA1c, respectively.

**Conclusion:**

Circulating TF^+^PS^+^MPs and those derived from platelet, lymphocytes and endothelial cells were elevated in patients with T1DM.

## Introduction

Type 1 diabetes mellitus (T1DM) is a multifactorial autoimmune disease characterized by destruction of pancreatic beta cells.^1^ Patients with T1DM have to use exogenous insulin replacement from diagnosis and face the risks of developing micro- and macro-vascular complications, potentially leading to a poor quality of life and premature mortality.^2^,^3^

Procoagulant microparticles (MPs) are heterogeneous populations of small vesicles which express phosphatidylserine (PS) ranging in size from 0.1 to 1.0μm, which are larger and more heterogeneous than exosomes (30 to 100nm).^4^ They can be released from most types of cells including erythrocytes, platelets, lymphocytes and endothelial cells during their activation, injury, or apoptosis.^5^ MPs are recognized multifunctional structures containing proteins, genetic information and lipids, which facilitate cross-talk between cells and regulate various pathological conditions such as coagulation, vascular inflammation, endothelial dysfunction, angiogenesis, cell apoptosis and immune response.^6^,^7^ Circulating MPs are reported to be elevated in diseases like hypertension, stroke, coronary heart disease and metabolic syndrome.^8^–^11^ Accumulating data indicates that counts of MPs are increased both in diabetic animal models and in diabetic patients.^12^–^16^ Our previous meta-analysis indicates that total MPs, platelet-derived MPs (PMPs), monocyte-derived MPs (MMPs) and endothelium-derived MPs (EMPs) are significantly higher in patients with type 2 diabetes than those in controls.^17^ MPs derived from platelets, monocytes, and endothelial cells in diabetic patients with vascular complications are reported to be higher than in those without vascular complications.^18^–^22^ Previous studies also indicated increased counts of total MPs, PMPs and EMPs were in patients with T1DM.^18^,^23^

The procoagulant activity of MPs is mainly driven by the PS expression.^24^ The tissue factor (TF) present on the procoagulant MPs (TF^+^PS^+^MPs) activates both Factor IX (FIX) and Factor X (FX) to initiate coagulation by binding Factor VII/Factor VIIa (FVII/FVIIa) as a receptor,^25^ which greatly increases the procoagulant activity of PS^+^MPs.^26^ TF^+^PS^+^MPs facilitate the formation of thrombus by doubling their counts and expressing thrombotic molecules in a short time.^27^,^28^ Given the strong procoagulant activity of TF^+^PS^+^MPs, we hypothesized that they may be elevated as a consequence of developing T1DM diabetes and its associated complications.

In this study, we aim to investigate the circulating TF^+^PS^+^MPs and their subtypes derived from different cell types in patients with T1DM and healthy volunteers using multicolor flow cytometric assay and explore the potential association between TF^+^PS^+^MPs and the laboratory and clinical features.

## Materials and Methods

### Subjects

The T1DM patients and age and sex-matched healthy volunteers were recruited separately in this case-control study. The T1DM patients were from COntinuous Management and Biomarker Study of type 1 diabetes (COMBS-1) study, which is a single-center cohort study of T1DM since 2015. The COMBS-1 study continuously recruited patients with T1DM from the inpatient and outpatient department in West China Hospital, Sichuan University and peer groups for patients with diabetes who met the following criteria: (1) diagnosis of T1DM requires all the following criteria: a. meeting the criteria for the diagnosis of diabetes mellitus according to WHO 1999 Criteria;^29^ b. with any overt diabetes-related symptoms when onset (ie, thirsty, polyuria, polydipsia, polyphagia or weight loss); c. with the history of diabetic ketosis or ketoacidosis; d. needing long-term insulin treatment from diagnosis; e. body mass index (BMI) ≤25Kg/m^2^ at the onset of diabetes; f. the age of onset is less than 30 years; (2) over 14 years of age when recruitment. The exclusion criteria included: (1) known or confirmed mitochondrial diabetes and monogenic diabetes; (2) life expectancy shorter than 2 years; (3) patients with severe mental disorders or patients who are unable to cooperate with the follow-up; (4) pregnancy when recruitment; (5) patients who are not willing to follow the study protocol. Previous medical history data, vital signs, physical examinations and questionnaires of each patient were collected at baseline. Islet cell antibodies were not included in the recruitment criteria for the study as Chinese patients display less antibody positivity than Caucasian populations (reported 60.7% based on literature).^30^ In the current study, we further exclude patients meeting the following criteria: (1) overt vascular or hematological disease, thromboembolic or coagulation disorders; (2) treatment with aspirin, clopidogrel or anticoagulant drugs in the past four weeks. Age- and sex- matched healthy volunteers were recruited from the staffs and medical students in West China Hospital during the study period, if they were free of self-reported diabetes, hypertension, autoimmune disease, acute or chronic inflammation or disease related to thrombosis and hemostasis. All subjects gave written informed consent in accordance with local ethics committee recommendations before study enrollment. The study protocol was conducted in accordance with the Declaration of Helsinki, was approved by the ethics committee of West China Hospital, Sichuan University.

### Laboratory Tests

Routine blood cell count was detected by automated hematology analyzer. Fasting blood glucose (FBG), total cholesterol (TC), triglycerides (TG), creatinine were measured on an automatic biochemistry analyzer (Modular P800, Roche Diagnostics GmbH, Germany) according to standard laboratory procedures. HbA1c was determined by a method based on high-performance liquid chromatography (HPLC) which was approved by the National Glycohemoglobin Standardization Program (NGSP) (HLC-723 G8, Tosoh Corporation, Japan). Urinary albumin and creatinine were used to calculate urinary albumin-to-creatinine (UACR). High-sensitivity C-reactive protein (hs-CRP) was measured by latex immunoturbidimetric method. Dilated retinal photography was taken in all patients which were reviewed by an ophthalmologist (Jiaying Zhang). The diabetic retinopathy (DR) was diagnosed and graded based on the International Clinical Diabetic Retinopathy and Macular Edema Disease Severity Scale.^31^

### Isolation of Plasma MP

Blood was collected into sodium citrate tubes and was centrifuged at 2500×*g* for 10 mins at 20°C. The supernatant was then centrifuged again at 2500×*g* for 10 mins to obtain platelet-free plasma (PFP). The samples were then stored at −80°C until analysis.

### Immunolabelling of MPs

After thawing, 5μL of PFP was diluted to 50μL with phosphate-buffered saline (PBS). The samples were incubated with mAbs as follows in the dark for 30 mins at room temperature. Annexin-V-APC and PE-conjugated mAb against TF was used to mark total PS^+^MPs and TF^+^MPs, respectively. FITC-conjugated mAb against platelet glycoprotein GPIIbIII (FITC-CD41a) was used to label platelet-derived MPs (PMPs). BV421-conjugated mAb CD235a was used to identify red blood cell-derived MPs (RMPs). APC-Cy7-conjugated mAb CD3 and PerCp-Cy5.5-conjugated mAb CD20 were identified T lymphocytes-derived MPs (TMPs) and B lymphocytes-derived MPs (BMPs), respectively. PE-CF594-conjugated mAb against CD14 was used to identify MMPs. PE-Cy7-conjugated mAb against VE-Cadherin (CD144) and V510-conjugated mAb against V-CAM1 (CD106) were used to identify EMPs. All reagents were purchased at BD Bioscience (San Diego, CA, USA). After incubation, 100μL of binding buffer was added. To determine the concentrations of the TF^+^MPs, 5.0 μL Flow-Count Fluorospheres (Beckman Coulter Immunotech, USA) was added to each tube. Samples were then prepared for flow cytometric analysis.

### Flow Cytometric Analysis

**Figure 1:**
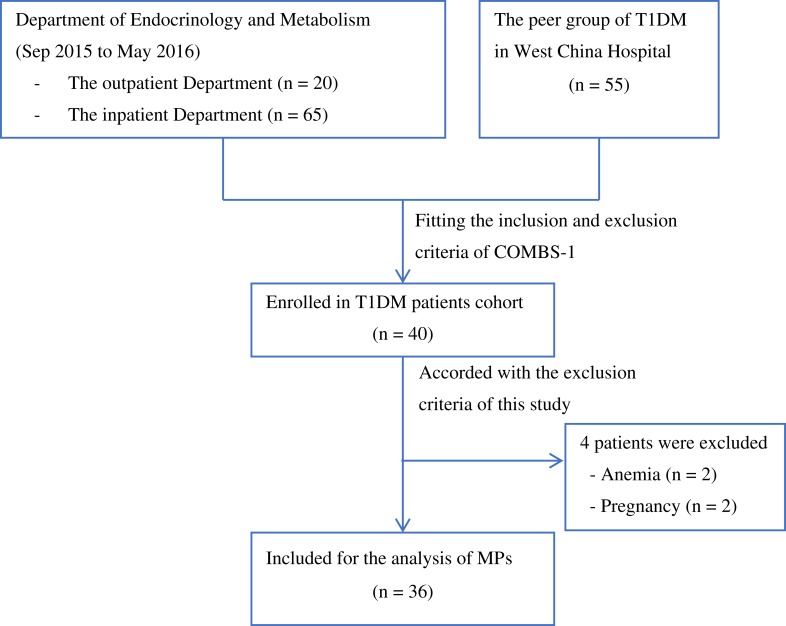
Flowchart of T1DM patient enrollment.

The prepared samples were detected using established protocol in the FACSAria cytometer (Becton Dickinson, San Jose, CA, USA) equipped with the FACS Diva 5.0 software and data were analyzed by FlowJo 10 (Tree Star, Ashland, OR, USA).^32^ MPs were analyzed based on their parameters of size and fluorescence. Firstly, the upper and lower limits of the MPs were determined on the size using 1.0μm calibration beads and 0.1μm calibration beads (Nano Fluorescent Size Standard Kit, Spherotech, USA). As shown in Figure 1, the events ranged in this gate and combined with positive Annexin-V expression (label for PS) were identified as procoagulant MPs (PS^+^MPs). Secondly, different types of PS^+^MPs were further distinguished by surface markers from the originated cells. To calculate the absolute value from each sample, flow-count fluorosperes were introduced since the total number of microspheres present in each sample was known. The count of MPs was calculated using the following formula:

$${\rm{Absolute\,count\,of\,MPs\,}}\left({{\rm{Beads}}/{\rm{\mu\,L}}} \right) = {\rm{Number\,of\,MPs\, counted\,}}\left({{\rm{Beads}}/{\rm{\mu\,L}}} \right) \times {\ }{{{\rm{Flow\,{\hbox-}\,count\,fluorosperes\,assayed \,MPs\,}}\left({{\rm{Beads}}/{\rm{\mu\,L}}} \right)} \over {{\rm{Number\,of\,fluorosperes\,counted\,MPs\,}}\left({{\rm{Beads}}/{\rm{\mu\,L}}} \right)}}$$

The ratio of TF^+^PS^+^MPs and PS^+^MPs (TF^+^PS^+^MPs/PS^+^MPs) was calculated by dividing TF^+^PS^+^MPs by PS^+^MPs.

### Statistical Analyses

Distribution of the data was tested by the Kolmogorov–Smirnov test. Continuous variables were presented as means ± standard deviations (SDs) when normally distributed or medians and ranges when not. Difference between groups was analyzed by paired student’s *t*-test for comparing normally distributed continuous data and Mann–Whitney *U*-test for those non-normally distributed data. Pearson’s correlation analysis and Spearman correlation analysis were performed to assess the correlation between MPs and clinical as well as laboratory variables among T1DM patients for normally and non-normally distributed data, respectively. Among the patients with T1DM, two subgroup analyses were conducted based on the presentation of DR and glucose control (HbA1c over or not over 7%). A 2-tailed *p* <0.05 was considered as statistical significant. Analyses were performed with SPSS 19.0 (Spss, Inc., Chicago, IL).

## Results

### Clinical Characteristics

The flowchart of patient recruitment is shown in Figure 1. We included 36 patients with T1DM and 36 age and sex-matched healthy volunteers in the study. The baseline characteristics of T1DM patients and healthy controls are presented in Table 1. The median age of T1DM patients was 23.5 (range from 16.0 to 46.0) years and 24 patients (67%) were females. The median duration of diabetes was 4.8 (ranged from 0.1 to 26.8) years. All T1DM patients were treated with insulin, however, 22 of 36 patients (61%) were noted to have an HbA1c above 7%. As shown in Table 1, the waist-hip ratio (WHR), TG, HbA1c, FBG, UACR and hs-CRP were higher in T1DM patients compared with healthy controls. There were no significant differences in age, blood pressure, blood cell counts or total cholesterol between the two groups. DR was identified in 16 (44%) of the T1DM patients, among which 15 of them had non-proliferative diabetic retinopathy (9 mild and 6 moderate) and one had proliferative diabetic retinopathy. None of the studied patients had albuminuria (defined as UACR >30 mg/g) or macro-vascular complications or cardiovascular diseases.

**Table 1:** Clinical Characteristic of Patients with T1DM and Healthy Controls. Data are Presented as Means ± Standard Deviations (SDs) or Medians (Ranges)

Variable	T1DM	Healthy Control	*P* value
N	36	36	–
Male/Female	12/24	12/24	–
Median age (yrs)	23.5 (16.0–46.0)	24.5 (19.0–45.0)	0.197
Duration of diabetes (yrs)	4.8 (0.1–26.8)	–	–
BMI (kg/m^2^)	21.2 ± 2.3	20.6 ± 1.8	0.640
WHR	0.88 ± 0.05	0.84 ± 0.05	0.001
SBP (mmHg) DBP (mmHg)	107.9 ± 8.4 67.2 ± 7.1	111.5 ± 7.3 69.6 ± 7.0	0.056 0.146
RBC (×10^12^/L)	4.5 ± 0.5	4.4 ± 0.5	0.254
WBC (×10^9^/L)	5.7 ± 1.7	5.8 ± 1.6	0.711
Neutrophilia (×10^9^/L)	3.0 ± 0.8	3.5 ± 1.3	0.096
Lymphocyte (×10^9^/L)	1.8 ± 0.5	1.7 ± 0.4	0.639
Platelet (×10^9^/L)	182.0 ± 61.2	208.0 ± 49.5	0.076
Total cholesterol (mmol/L)	4.62 ± 1.0	4.30 ± 0.76	0.137
LDL-C (mmol/L)	2.43 ± 0.71	2.23 ± 0.68	0.231
HDL-C (mmol/L)	1.64 ± 0.44	1.58 ± 0.33	0.459
Triglycerides (mmol/L)	1.01 ± 0.46	0.85 ± 0.39	0.053
FBG (mmol/L)	11.40 ± 4.72	4.65 ± 0.45	<0.001
HbA1c (%)	8.58 ± 2.43	4.98 ± 0.34	<0.001
Serum creatinine (umol/L)	60.7 ± 11.5	61.0 ± 10.0	0.944
UACR	6.60 (2.60–23.05)	5.55 (2.80–8.80)	0.020
hs-CRP (mg/dL)	1.21 ± 0.50	0.28 ± 0.13	<0.001
Diabetic retinopathy	16/36	-	-
NPDR	15/16	-	-
PDR	1/16	-	-

**Abbreviations:** T1DM, type 1 diabetes mellitus; BMI, body mass index; WHR, waist-hip-ratio; SBP, systolic blood pressure; DBP, diastolic blood pressure; RBC, red blood count; WBC, white blood count; LDL-C, low-density lipoprotein cholesterol; HDL-C, high-density lipoprotein cholesterol; FBG, fasting blood glucose; HbA1c, glycated hemoglobin; UACR, urinary albumin-to-creatinine ratio; hs-CRP, highly sensitive C-reactive protein; NPDR, non-proliferative diabetic retinopathy; PDR, proliferative diabetic retinopathy

### Quantification of PS^+^MPs in Two Groups

**Figure 2:**
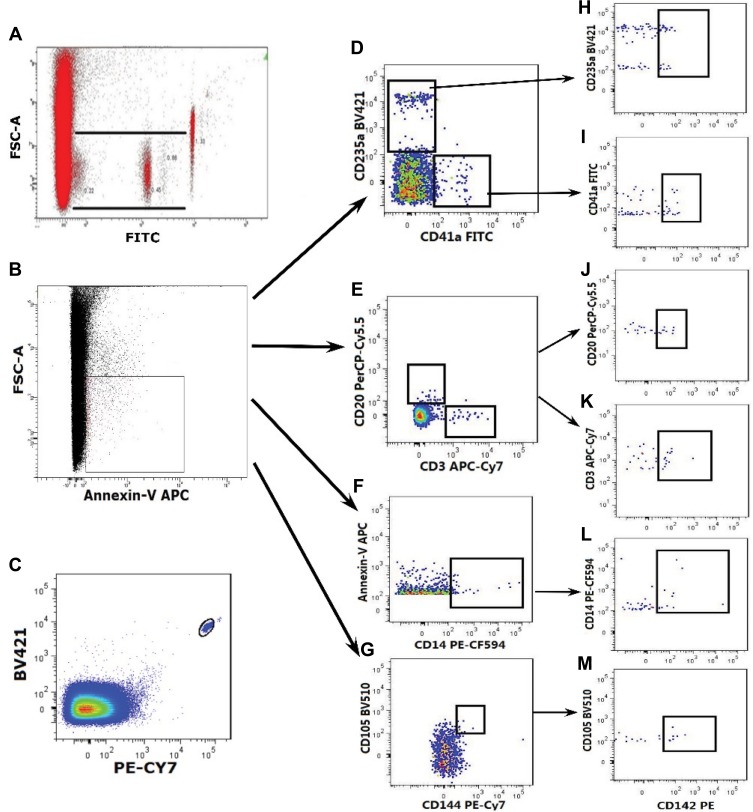
Quantitative detection of PS^+^MPs and TF^+^PS^+^MPs by Flow cytometry. Quantitative detection of PS^+^MPs and TF^+^PS^+^MPs by Flow cytometry. (**A**) Events ranged from 0.1μm to 1μm in size using Nano Fluorescent Size Standards; (**B**) Events ranged from 0.1 μm to 1.0 μm and binding annexin V were considered as PS^+^MPs; (**C**) Flow-Count Fluorospheres were gated on BV421/PE-Cy7 dot plot; (**D**) Gate of PS^+^MPs derived from platelets and erythrocytes (PS^+^PMPs and PS^+^RMPs, respectively); (**E**) Gate of PS^+^MPs derived from T cells and B cells (PS^+^TMPs and PS^+^BMPs, respectively); (**F**) Gate of PS^+^MPs derived from monocytes (PS^+^MMP); (**G**) Gate of PS^+^MPs derived from endothelium cells (PS^+^EMPs); (**H**) Gate of TF^+^PS^+^RMPs; (**I**) Gate of TF^+^PS^+^PMPs; (**J**) Gate of TF^+^PS^+^BMPs; (**K**) Gate of TF^+^PS^+^TMPs; (**L**) Gate of TF^+^PS^+^MMPs; (**M**) Gate of TF^+^PS^+^EMPs.

**Figure 3:**
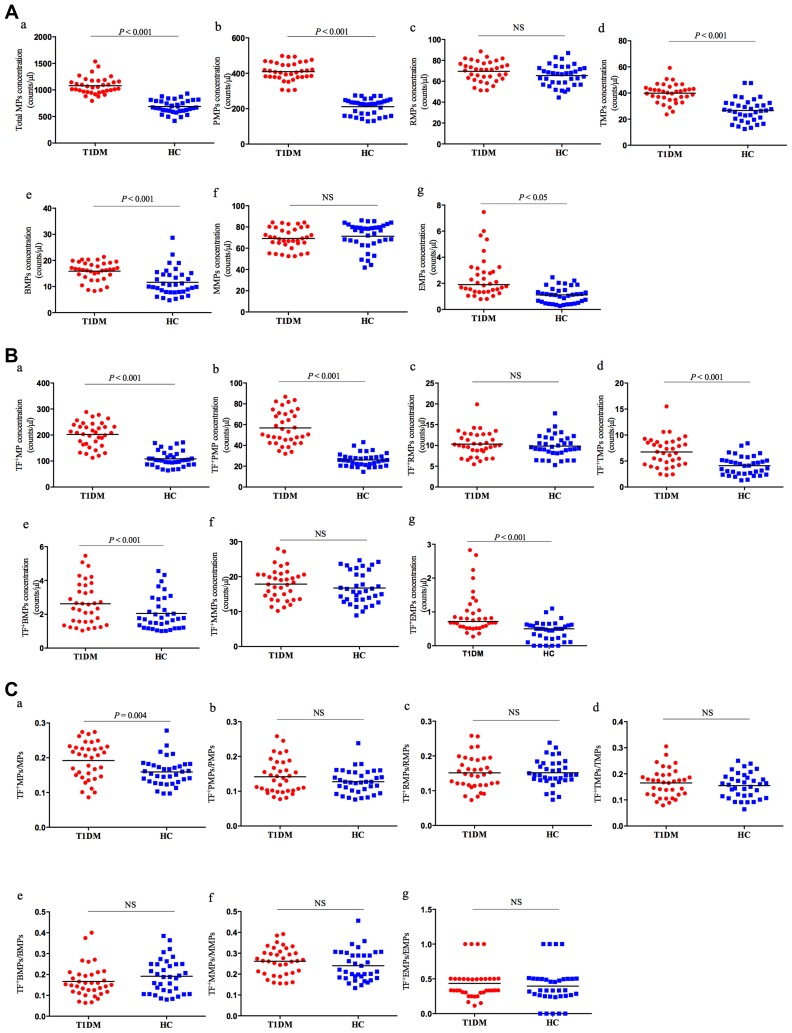
The counts of PS^+^MPs and TF^+^PS^+^MPs in T1DM patients and healthy controls. (**A**). Flow cytometry analysis of PS^+^MPs in T1DM patients and healthy controls. (a) total PS^+^MPs; (b) PMPs; (c) RMPs; (d) TMPs; (e) BMPs; (f) MMPs and (g) EMPs. (**B**) Flow cytometry analysis of TF^+^MPs in T1DM patients and healthy controls. (a) total TF^+^MPs; (b) TF^+^PMPs; (c) TF^+^RMPs; (d) TF^+^TMPs; (e) TF^+^BMPs; (f) TF^+^MMPs and (g) TF^+^EMPs. (**C**) The ratio of TF^+^MPs and PS^+^MPs (TF^+^MPs/PS^+^MPs) in T1DM patients and healthy controls. (a). TF^+^PS^+^MPs/PS^+^MPs; (b) TF^+^PS^+^PMPs/PS^+^PMPs; (c) TF^+^ PS^+^RMPs/PS^+^RMPs; (d) TF^+^PS^+^TMPs/PS^+^TMPs; (e) TF^+^PS^+^BMPs/PS^+^BMPs; (f) TF^+^PS^+^MMPs/PS^+^MMPs and (g) TF^+^PS^+^EMPs/PS^+^EMPs. Data are expressed as MPs counts per microliter of PFP. **Abbreviations:** PS, phosphatidylserine; MPs, microparticles; PMPs, platelet-derived MPs; TF, tissue factor; RMPs, red blood cell-derived MPs; TMPs, T lymphocytes-derived MPs; BMPs, B lymphocytes-derived MPs; MMPs, monocytes-derived MPs; EMPs, endothelium-derived MPs.

We identified the plasma MPs using flow cytometry by gating on particle size (ranging from 0.1μm to 1μm) with positive annexin V and with specific antibodies (Figure 2). As shown in Figure 3A, we found that the counts of total PS^+^MPs in T1DM patients were elevated compared with healthy controls (1078.5 ± 158.08 vs 686.84 ± 122.04/μL; *P* < 0.001). There was increased counts of PMPs (409.47 ± 52.03 vs 211.18 ± 45.44/μL; *P* <0.001), TMPs (39.89 ± 7.19 vs 26.66 ± 8.71/μL; *P* <0.001) and BMPs (15.89 ± 3.57 vs 11.61 ± 5.18/μL; *P* <0.001) in T1DM patients compared to the controls. The EMP levels identified by expression of CD144 and CD105 were markedly elevated in the patients with the T1DM compared with the controls (2.47 ± 1.59 vs 1.08 ± 0.61/μL; *P* <0.001). There were no significant differences between T1DM patients and healthy controls in circulating levels of RMPs (29.73 ± 9.82 vs 31.93 ± 8.86/μL; *P* = 0.32) or MMPs (69.18 ± 10.05 vs 71.30 ± 12.45/μL; *P* = 0.43).

### Quantification of TF^+^PS^+^MP in Two Groups

As shown in Figure 3B, TF^+^PS^+^MP counts were greater for the T1DM patients than healthy controls (202.10 ± 47.47 vs 108.33 ± 29.42/μL; *P* <0.001), mostly derived from platelets (56.76 ± 15.95 vs 26.03 ± 6.30/μL; *P* <0.001), T cells (6.74 ± 2.81 vs 4.11 ± 1.81/μL; *P* <0.001), B cells (2.62 ± 1.24 vs 2.05 ± 0.99/uL; *P* = 0.034) and endothelial cells [0.72 (0.27–2.83) vs 0.50 (0.00–1.10), *P* <0.001]. The TF^+^PS^+^MPs/PS^+^MPs were also elevated in the T1DM patients (0.16 ± 0.04 vs 0.19 ± 0.05, P = 0.004, Figure 3C). However, none of the ratios of cell-origin specific TF^+^PS^+^MPs were statistically significant. There were no significant differences between T1DM patients and healthy controls in TF^+^PS^+^MPs derived from erythrocytes (10.31 ± 2.93 vs 9.84 ± 2.69/μL, *P* = 0.47) or monocytes (17.86 ± 4.42 vs 16.73 ± 4.54/μL, *P* = 0.28).

### Subgroup Analysis of MPs in Patients with T1DM

T1DM patients were divided into two groups based on the presence of DR. As presented in Table 2, patients with DR had a greater concentration of TF^+^PS^+^MP than those without DR (*P* <0.001). There were no differences between these two groups in PS^+^MPs, and other types of TF^+^PS^+^MPs. Subgroup analysis stratified HbA1c was also performed in patients with T1DM. Patients with HbA1c higher than 7% had a greater count of TF^+^PS^+^MPs derived from platelet than those with HbA1c less than 7% (*P* <0.001).

**Table 2:** Sub-Analysis of PS^+^MPs and TF^+^PS^+^MPs in T1DM Patients According to Glucose Control and DR. Data are Presented as Mean ± SDs

Variable (Counts/μL)	DR			HbA1c		
with DR (n = 16)	Without DR (n = 20)	*P* value	HbA1c >7 (n = 22)	HbA1c ≤ 7 (n = 14)	*P* value
PS^+^MPs	1041.27 ± 151.26	1108.29 ± 160.83	0.211	1081.75 ± 164.51	1072.03 ± 151.16	0.893
TF^+^MPs	236.17 ± 29.24	174.85 ± 41.44	<0.001	197.54 ± 44.91	211.22 ± 53.09	0.365
PMPs	393.53 ± 57.59	422.22 ± 44.54	0.101	406.45 ± 51.89	415.50 ± 54.08	0.615
TF^+^PMPs	60.55 ± 16.78	53.73 ± 15.00	0.207	64.03 ± 14.40	42.24 ± 5.57	<0.001
RMPs	69.15 ± 9.60	69.87 ± 9.73	0.825	68.93 ± 10.14	70.78 ± 8.48	0.590
TF^+^RMPs	9.76 ± 2.26	10.76 ± 3.37	0.317	10.50 ± 2.96	9.94 ± 2.97	0.599
TMPs	40.81 ± 7.78	39.16 ± 6.79	0.500	40.41 ± 7.87	38.87 ± 5.78	0.552
TF^+^TMPs	6.19 ± 2.45	7.19 ± 3.06	0.295	6.91 ± 2.93	6.40 ± 2.66	0.611
BMPs	14.98 ± 3.74	16.62 ± 3.35	0.175	15.84 ± 3.66	15.99 ± 3.53	0.907
TF^+^BMPs	2.43 ± 1.35	2.77 ± 1.16	0.427	2.79 ± 1.29	2.29 ± 1.13	0.263
MMP	66.88 ± 8.26	71.01 ± 11.14	0.225	70.89 ± 9.80	65.74 ± 10.06	0.150
TF^+^MMP	16.60 ± 3.84	18.87 ± 4.69	0.128	18.20 ± 5.09	17.18 ± 2.73	0.519
EMPs	2.24 ± 1.49	2.65 ± 1.68	0.452	2.44 ± 1.53	2.52 ± 1.76	0.886
TF^+^EMPs	0.98 ± 0.73	0.91 ± 0.53	0.749	0.99 ± 0.67	0.84 ± 0.50	0.510

**Abbreviations:** T1DM, type 1 diabetes mellitus; DR, diabetic retinopathy; HbA1c, glycated hemoglobin; PS, phosphatidylserine; MPs, microparticles; PMPs, platelet-derived MPs; TF, tissue factor; RMPs, red blood cell-derived MPs; TMPs, T lymphocytes-derived MPs; BMPs, B lymphocytes-derived MPs; MMPs, monocytes-derived MPs; EMPs, endothelium-derived MPs.

### Correlations of MPs with Clinical Parameters in Patients with T1DM

The correlative analyses showed limited significant associations with the PS^+^MPs or TF^+^PS^+^MPs, expect some isolated findings (Supplementary Tables 1–7).

## Discussion

Our study suggests that the circulating procoagulant PS^+^MPs and those derived from platelets, lymphocytes and endothelium, as well as their TF expressing forms, were elevated in patients with T1DM compared to healthy controls. To our knowledge, this is the first study investigating the TF^+^PS^+^MPs in T1DM patients.

TF is a procoagulant protein expressed constitutively on the membrane of most non-vascular cells and expressed inductively on monocytes and endothelial cells.^33^,^34^ The expression of functional TF on MPs could significantly enhance their procoagulant activity in addition to PS.^26^,^35^–^37^ A recent study suggested that the TF^+^ and PS^+^ MPs were associated with meal intake, and lipid-lowering agents in T1DM patients.^38^ In our study, the elevated counts of TF^+^MPs in patients with T1DM, mostly derived from platelet, lymphocytes and endothelial cells, suggested a hypercoagulable state of T1DM patients.^39^ TF^+^PS^+^MP count was higher in patients with established microvascular complications without an obvious signal of its subgroup. It suggests TF^+^PS^+^MP may reflect overall damage of the blood components including circulating cells and epithelium. We also observed elevated TF^+^PS^+^PMP count in patients with higher HbA1c. It is partially because the platelet contributes to the highest count of MPs in the circulation, and also suggests that platelet and TF may be more sensitive to hyperglycemia compared with other blood cells.^40^,^41^

Our results of the PS^+^MPs were in line with previous studies.^18^,^23^ Sabatier et al^18^ suggested the PS^+^MPs, PS^+^PMPs and PS^+^EMPs increased in patients with T1DM, while only PS^+^MPs increased in patients with type 2 diabetes. Salem et al^23^ showed an elevation of PS^+^PMPs in patients with T1DM, especially those with microalbuminuria or other complications, and suggested the PS^+^PMPs could be a biomarker of microvascular complications in T1DM patients. Bergen et al^42^ reported an elevation of total unlabeled MPs and PS^+^MPs in T1DM patients, especially in patients with microvascular complications. Our study further suggested the TF^+^ subsets of total PS^+^MPs, PS^+^PMPs and PS^+^EMPs were also elevated in the patients with T1DM, and may be potential biomarkers of the disease and its complications. The strength of MPs and TF^+^MPs as biomarkers of T1DM is that they are not strongly confounding with traditional risk factors of diabetes as suggested in our study. However, more studies with larger sample size and different controls are needed before their clinical application.

EMP is a small portion of the circulating PS^+^MPs, associating with functional status and stability of endothelial cell.^43^ Multiple in vivo and in vitro studies showed the EMPs contribute to coagulation, angiogenesis, vertebral capillary damage and other vascular effects. Increased EMP levels were found in several pathological conditions such as hypertension, dyslipidemia, atherosclerosis, metabolic syndrome and coronary artery disease.^8^,^11^,^44^–^47^ Sabatier et al^18^ have shown that the levels of EMPs were elevated in T1DM and associated with microvascular complications, suggesting that EMPs could be a marker of diabetes-related vascular disease. However, our results did not show an obvious elevation of EMP level in patients with DR. It could be because most of our patients with DR had only mild or moderate nonproliferative DR but only one patient was identified with proliferative DR. And we did not identify other microvascular complications or cardiovascular co-morbidities among our patients.

This study presents some limitations. In this study, we use the immunological method to detect PS^+^MPs levels using specific fluorescence antibodies, but this method could not provide information about whether the TF^+^PS^+^MPs are functionally active. Besides, Our study was designed as a case-control study with limited sample size. We cannot conclude the causation or the pathophysiological link between the procoagulant MPs and the phenotypes. The results require validation in other ethnic populations.

In conclusion, we show that TF^+^PS^+^MPs from different origins increased in patients with T1DM. It indicated an abnormal procoagulant status in the T1DM patients. They may be potentially used as biomarkers of T1DM and its complications after further exploration and confirmation, and support investigation of the development of TF+MPs from different origins in the hyperglycemia
